# Retraction: Associations of TGFBR1 and TGFBR2 gene polymorphisms with the risk of hypospadias: a case-control study in a Chinese population

**DOI:** 10.1042/BSR-2017-0713_RET

**Published:** 2022-08-03

**Keywords:** Case-control, Gene polymorphism, Hypospadias, Risk, TGFBR1, TGFBR2

This article is being retracted from *Bioscience Reports* at the request of several listed co-authors who claim to have had no involvement in the paper and that they were unaware of its submission and publication. The corresponding author has been contacted with regards to the retraction and has not responded to the Journal's queries or the concerns raised. Several listed co-authors wish to retract the article and the Editorial Board agree with the Retraction.

